# The Effect of Therapeutic Adherence on the Effectiveness of a Digital Therapeutic Exercise Program: A Propensity Score Matching Analysis

**DOI:** 10.3390/healthcare11192614

**Published:** 2023-09-23

**Authors:** Markus Klingenberg, Andreas Elsner, Jan-Steffen Pooth, Felix Patricius Hans, Leo Benning

**Affiliations:** 1 Beta Klinik, 53227 Bonn, Germany; 2 German Institute of Orthopedics Osteopathy and Sports Medicine, 33604 Bielefeld, Germany; 3 University Emergency Center, Medical Center—University of Freiburg, 79106 Freiburg, Germany; 4 Faculty of Medicine, University of Freiburg, 79110 Freiburg, Germany

**Keywords:** digital therapeutics, movement exercise, digital health, musculoskeletal health

## Abstract

Nonspecific back pain (NSBP) contributes greatly to the overall burden of disease from musculoskeletal conditions. Digital therapeutics (DTx) aims to address the excess demand for movement and exercise therapy resulting from this spectrum of conditions. This study aims to investigate the differential therapeutic response of NSBP to different use profiles of a digital home exercise program. Methods: This study used a PSM model to comparatively assess the achievement of a clinically relevant pain improvement among patients who exhibit a high use (HU), intermediate use (IU), low use (LU), or sub-LU use profile. Sensitivity analyses with commonly accepted thresholds for clinically relevant improvements were conducted. Results: Higher use profiles show a higher probability of achieving a clinically relevant improvement of self-reported pain intensities. Additionally, the achievement of any higher use level is associated with a significant increase in the probability of achieving a clinically relevant improvement. Conclusion: To enable the optimal effectiveness of DTx home exercise programs, an HU use profile should be pursued. This finding is in line with earlier guidance for the achievement of optimal therapeutic benefit from conventional movement and exercise therapy and underscores the importance of a cross-disciplinary effort from patients, healthcare professionals and system stakeholders alike to maximize the therapeutic effect from DTx.

## 1. Background

Nonspecific musculoskeletal pain of the back is one of the leading drivers for burden of disease worldwide [1,2]. Beyond its impact on quality of life, it has significant effects on work absenteeism and therefore not only incurs high direct, but also indirect, healthcare costs [3]. These factors underscore the relevance of this condition for both individual patients and the healthcare systems serving them.

Beyond general therapeutic principles such as motivating patients to maintain an active lifestyle and providing condition-specific educational measures, the strongest therapeutic recommendation for the non-pharmacological management of nonspecific back pain consists of movement and exercise therapy, as outlined in international treatment guidelines [1,4]. Movement and exercise therapy becomes one of the central elements of the treatment when the symptoms persist and the pain evolves into a chronic medical condition (i.e., symptoms persisting for more than 12 weeks) [1,5].

As conventional movement and exercise therapy is typically administered by a qualified therapist [5], patients in need often encounter capacity shortages that lead to prolonged waiting times and, hence, delayed access to the required care. This has immediate effects on the adequate and timely therapy of patients with a broad spectrum of musculoskeletal conditions [6]. Beyond the challenges above, the authors see that limited capacities also increase the probability of an insufficient diagnostic workup, neglecting psychosocial, and other extravertebral factors that can sustain nonspecific musculoskeletal conditions.

Advances in the field of digital therapeutics (DTx) have opened up the opportunity to fill these capacity shortages with effective therapeutics that can be used independent of time and space and could therefore contribute to meeting the overall demand for care in the spectrum of conditions discussed above [7].

Yet, as with all long-term therapies, therapeutic adherence (i.e., the extent to which patients follow the jointly agreed upon therapeutic approach) has a significant effect on the clinical effectiveness of any DTx [8]. While the reasons for poor adherence are manifold and have been studied extensively before [9,10,11] this study aims to quantify the effect of different extents of therapeutic adherence on the effectiveness of the DTx ViViRA. ViViRA is a digital therapeutic exercise therapy available upon prescription under the legal DTx framework of the German healthcare system as a so-called Digitale Gesundheitsanwendung (DiGA) [12]. The purpose of this study, therefore, is to provide insights into the relevance of therapeutic adherence for the achievement of a therapeutic benefit from DTx delivering a digital movement and exercise therapy. The hypothesis of this study is that the maintenance of a recommended therapeutic adherence has a higher probability of leading to a clinically relevant improvement in pain symptoms than any lower use profile.

## 2. Methods

**Figure 1:**
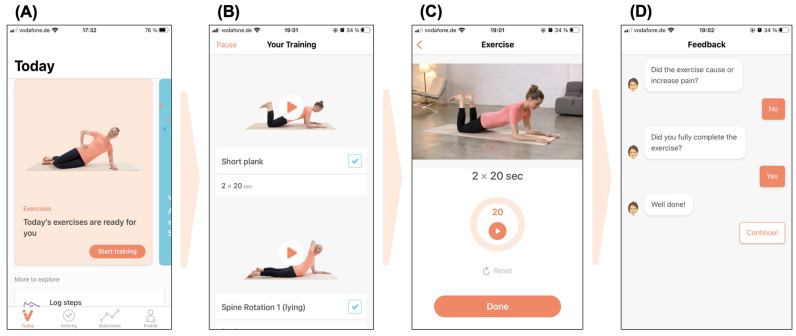
Patient interface. (**A**) ‘Today Screen’ with a prompt to start a daily routine and tabs to navigate progress, outcomes and settings; (**B**) selection of four exercises based on the onboarding assessment and continuous feedback, from which patients can select the exercises in their preferred order; (**C**) audio and video-based tutorial on the respective exercise and assistance for the correct execution of the exercise (e.g., time, exercise counts); (**D**) feedback on the completion of the exercise and adaptation of future exercises based on pain and functional limitations.

The data included in this study were collected during the preliminary market approval phase of the DTx ViViRA as a DiGA in the German healthcare system between 20 October 2020 and 17 February 2022. As a DiGA, ViViRA is a medical device classified as risk class I under the Medical Device Directive (MDD) and is available to the entire patient population of the statutory health insurance (SHI) system upon prescription by a qualified healthcare professional. Once the DiGA is activated, it provides a guided home exercise program that undergoes a stepwise personalization for each patient to account for progress, symptom development and potential movement limitations. A prescription is valid for 90 days and grants daily access to the full spectrum of functions of the DiGA, which extends beyond the therapeutic elements (e.g., exercises, feedback-based progression and virtual follow-up assessments) by providing educational content to improve the digital and health literacy of patients, as well as habit-building elements (e.g., reminders, praise and virtual appreciation for making therapeutic progress) to promote a physically active lifestyle. The recommended therapy consists of at least three routines per week with one routine consisting of four consecutive movement exercises. Patients are encouraged to complete all exercises of one routine in one session. All exercises and accompanying educational material are presented with text, video and audio guidance to enable unsupervised training. After the completion of every exercise, patients provide binary feedback on movement limitations and pain sensations, which drives the personalization of the exercise program. The progression algorithm maintains a sufficiently high training stimulus by increasing the intensity of the exercise (e.g., through more repetitions or longer exercise durations) first, before the complexity of the exercise increases (e.g., addition to the sequence of movements required to complete the exercise). Additionally, patients are prompted to report their current pain intensity on a verbal–numerical rating scale (VNRS) on a weekly basis, while the functional status (i.e., mobility, strength and coordination) is assessed each month. The VNRS employed is an 11-point scale that extends from 0 to 10 in five categories (0 = no pain; 1–3 = light pain; 4–6 = intermediate pain; 7–9 = severe pain; 10 = extreme pain) and is based on previous research for the assessment of non-malignant pain [13,14]. The functional status, however, was not analyzed for this study. Figure 1 illustrates the user interface and the patient-directed guidance for an example exercise.

Under the provision of article 4 of the DiGA regulation (DiGAV), the acquisition of performance and outcome data is allowed during the preliminary market approval phase once informed consent by the respective patient has been given. The regulation specifies that this consent can be obtained digitally. Patients received a prescription for ViViRA—and access to the digital movement and exercise therapy—at the sole discretion of a qualified healthcare professional once the respective patient was diagnosed with a nonspecific or degenerative back pain from the spectrum of medical conditions ViViRA has obtained market approval for (ICD-10-GM M42.0, M42.1, M42.9, M53.2, M53.8, M53.9, M54.4, M54.5, M54.6, M54.8, M54.9, M99.02, M99.03, M99.04, M99.82, M99.83, M99.84, M99.92, M99.93, M99.94). The manufacturer and the authors of this study, therefore, did not have any influence on the enrollment of patients. Hence, this analysis is based on real-world-use data. Ethics approval was obtained from the ethics committee of the University Medical Center, Freiburg on 5 April 2022 under the reference 22-1104-retro. The study was registered in the Deutsches Register Klinischer Studien (DRKS), a WHO-affiliated study registry, on 21 July 2022 under the reference DRKS00028920.

Inclusion criteria were an age of ≥18 years, completion of at least one exercise and submission of any pain intensity of >0/10 on the VNRS at baseline and least one pain intensity after the baseline assessment. Access to the DiGA was provided only after (a) a prescription was issued by a qualified healthcare professional with consecutive approval by the respective SHI, or (b) after a patient approached their SHI independently and received approval on the basis of a prior confirmation of the diagnosis addressed.

Use of the exercise program was stratified as either a high use (HU, ≥three routines per week), an intermediate use (IU, ≥two routines per week), or a low use (LU, ≥one routine per week) profile. Baseline demographics were compared between the use profiles (Table 1). The outcome of interest was assessed binarily as the achievement of a clinically relevant improvement in pain intensity, which was established at a 30% pain reduction in accordance with global consensus [15,16] (Table 2). This was performed by assessing the relative improvement from the initial VNRS to the last reported VNRS before termination of the therapy program. Sensitivity analyses were performed on the basis of other commonly accepted criteria for a clinically relevant benefit (i.e., a 1-point difference on a VNRS, as proposed by Leiva et al., and a 2-point difference on a VNRS, as proposed by Salaffi et al.) [17,18].

To account for potential confounding through patient characteristics in the observational data, we employed a propensity score (PS) matching approach, which has been described elsewhere [19]. In brief, a PS estimates the probability of a patient becoming subject to a specific exposure, conditional on a set of baseline covariates. Yet, the estimation of the PS does not account for the actual exposure. Consequently, a distribution of PS among exposed patients and a distribution among unexposed patients result. Matching patients with a similar PS, in turn, allows the matched comparison of exposed and unexposed with similar baseline covariates.

We estimated a PS using a logistic model based on the baseline covariates of gender, concomitant physical therapy and/or pain medication at baseline, and the chronicity of the pain at baseline (Table 3). As the submission of the variables included in the PS were mandatory during the onboarding process of patients, no missing variables were detected. The assumption of conditional independence was assumed to hold due to the use of independent baseline covariates only; a balanced distribution of covariates was assessed per quintile of the PS and is provided in Supplementary File S1. As a second qualifying assumption, a region of common support was identified graphically and is provided in Supplementary File S2. Propensity score matching was performed as a nearest-neighbor matching with *n* = 5 between exposed and unexposed in each stratum. Sampling with replacement was used. Primary results were adjusted for multiple testing using the Bonferroni method. All calculations were performed in Stata 17.

The reporting in this article follows the adjustment of the STROBE guidelines for PS-based analyses proposed by Yao et al. [20], a reporting checklist is provided in Supplementary File S3.

## 3. Results

In total, 7628 patients who enrolled in the home exercise program for back pain between 20 October 2020 and 17 February 2022, completed at least one exercise and reported at least one pain score were included in this retrospective study. Owing to the automated data collection upon consent and activation of the DiGA, all potentially eligible patients could be included and analyzed. Overall, we see an overrepresentation of female patients in the sample (Table 1). While the majority of patients follow the treatment recommendation for at least three routines per week (i.e., HU profile), a relevant proportion of patients falls short of the recommended use (i.e., IU, LU and sub-LU) (Table 1). Yet, no clinically relevant differences in baseline pain intensity, chronicity of pain, concomitant use of pain medication and concomitant use of personal physical therapy could be detected (Table 1).

Prior to PS-matching, the probability of achieving a clinically relevant improvement in their pain intensity at the individual time of termination of use among patients with an HU profile is 39.2% (Table 2). IU, LU and sub-LU usage profiles are, in turn, associated with a relevantly reduced probability of achieving such an outcome (Table 2). Sensitivity analyses for more lenient thresholds of a clinically relevant improvement were conducted and showed 59.2% with a clinically relevant improvement among patients with HU, while patients with sub-LU achieved an improvement in 41.1% of the cases in the most lenient scenario (i.e., at least a 1-point VNRS improvement from baseline, Supplementary File S4). Applying a more conservative threshold for a clinically relevant improvement (i.e., at least a 2-point VNRS improvement from baseline), patients with HU achieved a relevant improvement in 41.3%, while patients with sub-LU profiles achieved a relevant improvement in 27.1% (Supplementary File S5). However, to assess how the increase in usage affects the probability of achieving a clinically relevant improvement in pain intensity independently, we performed a PS-matched analysis between HU and IU/LU/sub-LU, IU and LU/sub-LU and LU and sub-LU. As the submission of the variables included in the PS were mandatory during the onboarding process of patients, no missing variables were detected.

Patients with HU consecutively have a 9% (95%-CI 6.7–11.5%) higher probability of achieving a clinically relevant reduction in their pain intensity in comparison to all other use profiles (i.e., IU, LU, sub-LU) (Table 3). Similarly, patients with IU also have a 9% (95%-CI 5.5–12.6) higher probability of achieving a clinically relevant reduction when compared to patients with LU/sub-LU use profiles (Table 3). Achieving only a low use profile (i.e., LU), however, does not yield a significantly higher probability of meeting the criterion for a clinically relevant improvement in pain intensity when compared to the infrequent use of the program (i.e., sub-LU) (2%; 95%-CI −4.2–8.9%) (Table 3). These results imply that the achievement of any higher use level beyond LU yields a significantly higher probability of achieving a clinical benefit from the use of the exercise program, whereas an LU profile does not significantly increase the probability of such benefit beyond that of a sub-LU profile, where the latter equates to the mere sporadic use of the exercise program.

Sensitivity analyses were conducted with other commonly accepted thresholds for clinically relevant benefits. Both sensitivity analyses supported the results above and showed a significantly higher probability of achieving the respective clinically relevant improvement once patients adhered to IU or HU use profiles. Similar to the primary analysis, no significantly higher probability of a clinically significant benefit could be detected when comparing the LU use intensity and the sporadic (i.e., sub-LU) use profile. Tabulated results of the sensitivity analyses can be found in Supplementary Files S6 and S7.

## 4. Discussion

### 4.1. Primary Results

The main result of this analysis supports the primary hypothesis that the achievement of a clinically relevant improvement in pain intensity is dependent upon the maintenance of sufficient therapeutic adherence (Table 2). Specifically, maintaining the recommended HU profile is independently associated with a significantly higher probability of achieving a clinically relevant improvement when compared to any other (i.e., lower) use profile (Table 3). Yet, maintaining at least an IU profile is associated with a significantly higher probability of crossing the threshold of a clinically relevant improvement when compared to LU and sub-LU use profiles (Table 3). Interestingly, however, the maintenance of an LU profile does not yield a significant improvement over the sub-LU profile (Table 3). These findings are in line with previous studies and underscore the importance of a sufficiently high use frequency for movement and exercise therapy to yield clinically relevant results [10]. Sensitivity analyses with other commonly accepted thresholds for a clinically relevant improvement in pain symptoms were conducted and support these findings (Supplementary Files S4–S7).

For conventional movement and exercise therapy, an association between a sufficiently high therapeutic intensity (i.e., frequency of therapeutic exercises) and the therapeutic benefit has been well established [21,22]. As both conventional and digital movement and exercise therapy employ similar therapeutic principles (e.g., education, repeated and structured movement exercises and habit-building for an active lifestyle), it is a plausible finding that the same association exists for digital movement and exercise therapy for back pain. It highlights, however, that the effectiveness of a digital movement and exercise therapy is not exclusively and inherently dependent on the design and the functional composition of the respective DTx, but that it is greatly modulated by the extent of therapeutic adherence a patient can achieve throughout the use of the program.

### 4.2. Factors Influencing Therapeutic Adherence

Long-standing guidance from the World Health Organization (WHO) underscores that therapeutic adherence is “a primary determinant of the effectiveness of treatment, because poor adherence attenuates optimum clinical benefit” [9]. This is well in line with our findings, which illustrate a clear and incremental relationship between the use profiles and the probability of achieving a clinically relevant benefit (Table 3). However, what it is that enables patients to achieve higher use profiles remains unclear in our study. Previous research has found that a multitude of factors (i.e., health system-related, socioeconomic, therapy-related, patient-related and condition-related factors) influence adherence [9]. Although the first comprehensive reviews on adherence modulating features have been conducted [23], a systematic approach to identifying these factors in the context of DTx has not yet been established. Yet, the literature on adherence to DTx proposes a number of interventions that constitute therapy-related factors for improved adherence within the therapeutics itself. Firstly, the design of reminders and notifications offers the potential for improved adherence. Automatic reminders, as well as motivational and personalized messages, were associated with higher rates of adherence in multiple studies [11,24,25,26,27]. Secondly, social support has been shown to increase adherence [10,11,15,16,17,18,19,20,21,22,23,24,25,26,27]. As this proves difficult to implement as a feature in scalable DTx solutions, the field of human-like guidance through, for example, conversational agents has been proven to convey similar positive effects on therapeutic adherence [11]. Thirdly, monitoring of condition-specific (e.g., pain, range of movement and therapeutic progress) and wellbeing-associated metrics (e.g., mood, satisfaction) can provide important insights into which therapeutic prompt a patient would most likely interact with [11,26]. Lastly, the overarching concept of convenience has a notable effect. Generally, easy access, flexible use and short prompts are associated with higher adherence rates [28,29]. While the factors related to the therapeutics itself outlined above are not exhaustive, they offer a potential starting point for the incremental improvement in adherence to DTx.

Beyond these, however, the thorough exploration and consequent implementation of health system-related (e.g., training of healthcare professionals and the administrative processes of prescriptions and reimbursement), socioeconomic (e.g., ensuring that access to DTx is not dependent on the possession of a smartphone and that functionality is sustained on outdated hardware/software), patient-related (e.g., digital and health literacy is assessed and, if necessary, educational programs for the use of DTx alongside—or instead of—conventional therapies are established) and condition-related factors (e.g., detection of risk factors for progression of an unspecific or degenerative back pain into a chronic pain condition and variable focus on habit-building to support active lifestyles) is required to achieve the best possible benefit from DTx for both individual patients and the healthcare systems employing them.

### 4.3. Requirement of a System-Wide Effort for the Achievement of the Best Possible Therapeutic Benefit

We see that the required collaborative approach towards facilitating the best possible therapeutic adherence has not yet been pursued in the German healthcare system. Specifically, the notion of suboptimal therapeutic adherence as a unidimensional indicator for poor quality of the respective DTx, as put forward by some system stakeholders, defies the existing body of evidence on adherence and prevents the thorough integration of the novel field of DTx into the existing healthcare infrastructure [30,31]. Yet, from our perspective, these integrative efforts are of crucial importance to achieve the best possible benefit for both the patients and the healthcare system. The education of doctors and patients with regard to the characteristics and benefits of DTx is seen as a central enabler for integrating novel therapeutics into the healthcare system [32]. Ensuring, for example, an effective and efficient prescription and reimbursement process is seen as critical to facilitate doctor and patient access to DTx in a reimbursement-based healthcare system.

## 5. Limitations and Implications

The study at hand aims to assess the effect of higher frequencies of use of the DiGA ViViRA on its therapeutic effectiveness in reducing nonspecific back pain. While this effect could be demonstrated and while the findings are in line with prior research in this field [33,34,35], certain limitations apply to our study and warrant a careful interpretation. Firstly, the study is based on retrospective use data and therefore does not yield confirmatory power. Secondly, and due to the limitations of data that can be collected under the regulatory framework of the DiGAV, the collection of data was allowed only during the preliminary listing period of the DiGA ViViRA between 20 October 2020 and 17 February 2022. As the branch of DTx was newly introduced to the German healthcare system at this time, no steady state of patient characteristics can be assumed. Whether the use patterns and the adherence of patients has since evolved remains unclear. This limits the generalizability of the results presented. Likewise, the selection of patients who exhibited a certain use behavior at the time of enrollment (i.e., completed at least one exercise and reported at least one pain score) is a potential source of selection bias, which also impairs the generalizability of our work. While this restriction was required to assess the achievement of a clinically relevant benefit, it remains unclear whether systematic differences exist between patients who follow the prompt to report their pain intensities regularly and the patients who do not follow the prompt. Nonetheless, our work contributes the important insight that the therapeutic benefit of a digital movement and exercise therapy is highly dependent on the use profile of the individual patient. While this was demonstrated for conventional movement and exercise therapy long ago, the indication of the same dependency for digital movement and exercise therapy highlights the importance of a collaborative approach involving all stakeholders to enable the best possible benefit for patients.

## 6. Conclusions

This work presents a PS-matched analysis of the effect of different use profiles on the achievement of a clinically relevant pain improvement in patients with nonspecific back pain. While a positive effect of relatively higher use profiles on nonspecific musculoskeletal conditions in general and nonspecific back pain in particular has been reported for conventional movement and exercise therapy before, our work indicates a similar effect for DTx and DiGA. To achieve sufficiently high use profiles—corresponding to a sufficient therapeutic adherence—among patients under care with a DTx, however, a multi-stakeholder effort is required. As it was proposed for conventional therapeutics in a hallmark paper of the WHO decades ago, further research should revisit the five dimensions of adherence in the light of DTx in order to maximize the individual and system-wide benefits of such novel therapies.

## Figures and Tables

**Table 1:** Baseline demographics of patients with high use (HU), intermediate use (IU), low use (LU) and no relevant use profile (i.e., <1 routine/week, sub-LU).

	HU	IU	LU	Sub-LU
Patients, n (%)	4944 (64.8)	1358 (17.8)	1219 (16)	107 (1.4)
Age, years (SD)	47.35 (13.76)	44.76 (13.59)	44.49 (13.81)	46.47 (15.04)
Sex, % female	70.8	70.7	71.5	72.9
Pain intensity (SD)	5.44 (1.81)	5.5 (1.70)	5.47 (1.82)	5.96 (2.04)
Chronicity of pain, % chronic	67.5	67.4	68.2	70.1
Concomitant use of pain medication, %	26.2	28.1	26.6	31.8
Concomitant use of personal physical therapy, %	32.5	31.4	33.6	38.3

**Table 2:** Achievement of clinically relevant improvement in pain intensity prior to PS-matching for patients with high use (HU), intermediate use (IU), low use (LU) and no relevant use profile (i.e., <1 routine/week, sub-LU).

Usage Profile	Achievement of a Clinically Relevant Improvement in Pain Intensity at Termination of Use (%)
HU	39.2
IU	34.6
LU	26.5
Sub-LU	22.4

**Table 3:** Differential probability of achieving a clinically relevant improvement in pain intensity at the termination of use. PS-matched analysis for patients with high use (HU), intermediate use (IU), low use (LU) and no relevant use intensity (i.e., <1 routine/week, sub-LU). The Bonferroni method was used to adjust the results for multiple testing; significance can be assumed if *p* < 0.0167.

	*n*	Coefficient	Robust SE	z	*p* < |z|	95%-CI
HU vs. IU/LU/Sub-LU	7628	0.09	0.0124	7.34	<0.0001	0.067–0.115
IU vs. LU/Sub-LU	2684	0.09	0.019	4.85	<0.0001	0.054–0.126
LU vs. Sub-LU	1326	0.02	0.033	0.7	0.483	−0.042–0.089

## Data Availability

Primary data are not publicly available as their use is restricted through the German Digital Health Applications Regulation (DiGAV) Article 4. However, a meta-data framework can be made available upon reasonable request.

## Supplementary Materials

The following supporting information can be downloaded at: https://www.mdpi.com/article/10.3390/healthcare11192614/s1, File S1: Assessment of the distribution of each baseline covariate included in the estimation of the propensity scores (PS) between the exposed and unexposed patients across the quintiles of the PS., File S2: Graphical assessment of a region of common support of propensity scores (PS) for patients with high-intensity (hi), intermediate-intensity (ii), and low-intensity (li) use profiles., File S3: Reporting checklist for reporting PS-based analyses, Files S4–S7: Sensitivity analyses of commonly accepted thresholds for clinically relevant improvements in pain intensity. Reference [36] is cited in the supplementary materials.

## Abbreviations

DTx	Digital Therapeutics
DiGA	Digitale Gesundheitsanwendung (i.e., a DTx available upon prescription in the German healthcare system)
MDD	Medical Device Directive
SHI	Statutory Health Insurance
VNRS	Verbal Numerical Rating Scale
DiGAV	DiGA-Verordnung (i.e., legal framework for the market approval process of DiGA)
ICD-10-GM	International Classification of Diseases, 10th version, German Modification
DRKS	Deutsches Register Klinischer Studien (i.e., German Registry for Clinical Trials)
HU	High Use
IU	Intermediate Use
LU	Low Use
PS	Propensity Score
WHO	World Health Organization
