# Supplementary material for: The Effect of Therapeutic Adherence on the Effectiveness of a Digital Therapeutic Exercise Program: A Propensity Score Matching Analysis
Source: Healthcare (Basel). 2023 Sep 23;11(19):2614. doi: 10.3390/healthcare11192614 (PMC10572588; doi:10.3390/healthcare11192614)
Supplement: Supplementary file 1 [file healthcare-11-02614-s001.zip › Supplementary_1.pdf]

Supplementary File 1: Assessment of the distribution of each baseline covariate included in the estimation of the propensity scores (PS) between the exposed and unexposed patients across the quintiles of the PS.

```
. do "/var/folders/hz/9r6gkdps2lddy1b13x2kxkk80000gq/T//SD17798.000000"

. by quantile_hi treatment_binary_hi, sort: tabulate female

-------------------------------------------------------------------------------------------------------------------------------
-> quantile_hi = 1, treatment_binary_hi = 0

     female |      Freq.     Percent        Cum.
------------+-----------------------------------
          0 |        183       28.20       28.20
          1 |        466       71.80      100.00
------------+-----------------------------------
      Total |        649      100.00

-------------------------------------------------------------------------------------------------------------------------------
-> quantile_hi = 1, treatment_binary_hi = 1

     female |      Freq.     Percent        Cum.
------------+-----------------------------------
          0 |        240       27.27       27.27
          1 |        640       72.73      100.00
------------+-----------------------------------
      Total |        880      100.00

-------------------------------------------------------------------------------------------------------------------------------
-> quantile_hi = 2, treatment_binary_hi = 0

     female |      Freq.     Percent        Cum.
------------+-----------------------------------
          0 |        160       26.85       26.85
          1 |        436       73.15      100.00
------------+-----------------------------------
      Total |        596      100.00

-------------------------------------------------------------------------------------------------------------------------------
-> quantile_hi = 2, treatment_binary_hi = 1

     female |      Freq.     Percent        Cum.
------------+-----------------------------------
          0 |        295       31.75       31.75
          1 |        634       68.25      100.00
------------+-----------------------------------
      Total |        929      100.00

-------------------------------------------------------------------------------------------------------------------------------
-> quantile_hi = 3, treatment_binary_hi = 0

     female |      Freq.     Percent        Cum.
------------+-----------------------------------
          0 |        145       28.71       28.71
          1 |        360       71.29      100.00
------------+-----------------------------------
      Total |        505      100.00

-------------------------------------------------------------------------------------------------------------------------------
-> quantile_hi = 3, treatment_binary_hi = 1

     female |      Freq.     Percent        Cum.
------------+-----------------------------------
          0 |        294       28.82       28.82
          1 |        726       71.18      100.00
------------+-----------------------------------
      Total |      1,020      100.00
```

```
--------------------------------------------------------------------------------------------------------------------------------
-> quantile_hi = 4, treatment_binary_hi = 0

     female |      Freq.     Percent        Cum.
------------+-----------------------------------
          0 |        132       27.44       27.44
          1 |        349       72.56      100.00
------------+-----------------------------------
      Total |        481      100.00

--------------------------------------------------------------------------------------------------------------------------------
-> quantile_hi = 4, treatment_binary_hi = 1

     female |      Freq.     Percent        Cum.
------------+-----------------------------------
          0 |        271       25.81       25.81
          1 |        779       74.19      100.00
------------+-----------------------------------
      Total |      1,050      100.00

--------------------------------------------------------------------------------------------------------------------------------
-> quantile_hi = 5, treatment_binary_hi = 0

     female |      Freq.     Percent        Cum.
------------+-----------------------------------
          0 |        154       34.00       34.00
          1 |        299       66.00      100.00
------------+-----------------------------------
      Total |        453      100.00

--------------------------------------------------------------------------------------------------------------------------------
-> quantile_hi = 5, treatment_binary_hi = 1

     female |      Freq.     Percent        Cum.
------------+-----------------------------------
          0 |        343       32.21       32.21
          1 |        722       67.79      100.00
------------+-----------------------------------
      Total |      1,065      100.00


. by quantile_hi treatment_binary_hi, sort: tabulate CPT_baseline

--------------------------------------------------------------------------------------------------------------------------------
-> quantile_hi = 1, treatment_binary_hi = 0

CPT_baselin |
          e |      Freq.     Percent        Cum.
------------+-----------------------------------
          0 |        443       68.26       68.26
          1 |        206       31.74      100.00
------------+-----------------------------------
      Total |        649      100.00

--------------------------------------------------------------------------------------------------------------------------------
-> quantile_hi = 1, treatment_binary_hi = 1

CPT_baselin |
          e |      Freq.     Percent        Cum.
------------+-----------------------------------
          0 |        596       67.73       67.73
          1 |        284       32.27      100.00
------------+-----------------------------------
      Total |        880      100.00

--------------------------------------------------------------------------------------------------------------------------------
-> quantile_hi = 2, treatment_binary_hi = 0
```

```
CPT_baselin |
          e |      Freq.     Percent        Cum.
------------+-----------------------------------
          0 |        405       67.95       67.95
          1 |        191       32.05      100.00
------------+-----------------------------------
      Total |        596      100.00

-----------------------------------------------------------------------------------------------------------------------------------------
-> quantile_hi = 2, treatment_binary_hi = 1

CPT_baselin |
          e |      Freq.     Percent        Cum.
------------+-----------------------------------
          0 |        633       68.14       68.14
          1 |        296       31.86      100.00
------------+-----------------------------------
      Total |        929      100.00

-----------------------------------------------------------------------------------------------------------------------------------------
-> quantile_hi = 3, treatment_binary_hi = 0

CPT_baselin |
          e |      Freq.     Percent        Cum.
------------+-----------------------------------
          0 |        325       64.36       64.36
          1 |        180       35.64      100.00
------------+-----------------------------------
      Total |        505      100.00

-----------------------------------------------------------------------------------------------------------------------------------------
-> quantile_hi = 3, treatment_binary_hi = 1

CPT_baselin |
          e |      Freq.     Percent        Cum.
------------+-----------------------------------
          0 |        653       64.02       64.02
          1 |        367       35.98      100.00
------------+-----------------------------------
      Total |      1,020      100.00

-----------------------------------------------------------------------------------------------------------------------------------------
-> quantile_hi = 4, treatment_binary_hi = 0

CPT_baselin |
          e |      Freq.     Percent        Cum.
------------+-----------------------------------
          0 |        315       65.49       65.49
          1 |        166       34.51      100.00
------------+-----------------------------------
      Total |        481      100.00

-----------------------------------------------------------------------------------------------------------------------------------------
-> quantile_hi = 4, treatment_binary_hi = 1

CPT_baselin |
          e |      Freq.     Percent        Cum.
------------+-----------------------------------
          0 |        699       66.57       66.57
          1 |        351       33.43      100.00
------------+-----------------------------------
      Total |      1,050      100.00

-----------------------------------------------------------------------------------------------------------------------------------------
-> quantile_hi = 5, treatment_binary_hi = 0
```

```
CPT_baselin |
          e |      Freq.     Percent        Cum.
------------+-----------------------------------
          0 |        319       70.42       70.42
          1 |        134       29.58      100.00
------------+-----------------------------------
      Total |        453      100.00

-------------------------------------------------------------------------------------------------------------------------------
-> quantile_hi = 5, treatment_binary_hi = 1

CPT_baselin |
          e |      Freq.     Percent        Cum.
------------+-----------------------------------
          0 |        756       70.99       70.99
          1 |        309       29.01      100.00
------------+-----------------------------------
      Total |      1,065      100.00


. by quantile_hi treatment_binary_hi, sort: tabulate CPM_baseline

-------------------------------------------------------------------------------------------------------------------------------
-> quantile_hi = 1, treatment_binary_hi = 0

CPM_baselin |
          e |      Freq.     Percent        Cum.
------------+-----------------------------------
          0 |        433       66.72       66.72
          1 |        216       33.28      100.00
------------+-----------------------------------
      Total |        649      100.00

-------------------------------------------------------------------------------------------------------------------------------
-> quantile_hi = 1, treatment_binary_hi = 1

CPM_baselin |
          e |      Freq.     Percent        Cum.
------------+-----------------------------------
          0 |        591       67.16       67.16
          1 |        289       32.84      100.00
------------+-----------------------------------
      Total |        880      100.00

-------------------------------------------------------------------------------------------------------------------------------
-> quantile_hi = 2, treatment_binary_hi = 0

CPM_baselin |
          e |      Freq.     Percent        Cum.
------------+-----------------------------------
          0 |        424       71.14       71.14
          1 |        172       28.86      100.00
------------+-----------------------------------
      Total |        596      100.00

-------------------------------------------------------------------------------------------------------------------------------
-> quantile_hi = 2, treatment_binary_hi = 1

CPM_baselin |
          e |      Freq.     Percent        Cum.
------------+-----------------------------------
          0 |        674       72.55       72.55
          1 |        255       27.45      100.00
------------+-----------------------------------
      Total |        929      100.00

-------------------------------------------------------------------------------------------------------------------------------
```

-> quantile_hi = 3, treatment_binary_hi = 0

CPM_baseline	Freq.	Percent	Cum.
0	333	65.94	65.94
1	172	34.06	100.00
Total	505	100.00	

-> quantile_hi = 3, treatment_binary_hi = 1

CPM_baseline	Freq.	Percent	Cum.
0	686	67.25	67.25
1	334	32.75	100.00
Total	1,020	100.00	

-> quantile_hi = 4, treatment_binary_hi = 0

CPM_baseline	Freq.	Percent	Cum.
0	348	72.35	72.35
1	133	27.65	100.00
Total	481	100.00	

-> quantile_hi = 4, treatment_binary_hi = 1

CPM_baseline	Freq.	Percent	Cum.
0	758	72.19	72.19
1	292	27.81	100.00
Total	1,050	100.00	

-> quantile_hi = 5, treatment_binary_hi = 0

CPM_baseline	Freq.	Percent	Cum.
0	407	89.85	89.85
1	46	10.15	100.00
Total	453	100.00	

-> quantile_hi = 5, treatment_binary_hi = 1

CPM_baseline	Freq.	Percent	Cum.
0	940	88.26	88.26
1	125	11.74	100.00
Total	1,065	100.00	

. by quantile_hi treatment_binary_hi, sort: tabulate ChP_baseline

```
----------------------------------------------------------------------------------------------------------------------------
-> quantile_hi = 1, treatment_binary_hi = 0

ChP_baselin |
          e |      Freq.     Percent        Cum.
------------+-----------------------------------
          0 |        199       30.66       30.66
          1 |        450       69.34      100.00
------------+-----------------------------------
      Total |        649      100.00

----------------------------------------------------------------------------------------------------------------------------
-> quantile_hi = 1, treatment_binary_hi = 1

ChP_baselin |
          e |      Freq.     Percent        Cum.
------------+-----------------------------------
          0 |        257       29.20       29.20
          1 |        623       70.80      100.00
------------+-----------------------------------
      Total |        880      100.00

----------------------------------------------------------------------------------------------------------------------------
-> quantile_hi = 2, treatment_binary_hi = 0

ChP_baselin |
          e |      Freq.     Percent        Cum.
------------+-----------------------------------
          0 |        200       33.56       33.56
          1 |        396       66.44      100.00
------------+-----------------------------------
      Total |        596      100.00

----------------------------------------------------------------------------------------------------------------------------
-> quantile_hi = 2, treatment_binary_hi = 1

ChP_baselin |
          e |      Freq.     Percent        Cum.
------------+-----------------------------------
          0 |        315       33.91       33.91
          1 |        614       66.09      100.00
------------+-----------------------------------
      Total |        929      100.00

----------------------------------------------------------------------------------------------------------------------------
-> quantile_hi = 3, treatment_binary_hi = 0

ChP_baselin |
          e |      Freq.     Percent        Cum.
------------+-----------------------------------
          0 |        160       31.68       31.68
          1 |        345       68.32      100.00
------------+-----------------------------------
      Total |        505      100.00

----------------------------------------------------------------------------------------------------------------------------
-> quantile_hi = 3, treatment_binary_hi = 1

ChP_baselin |
          e |      Freq.     Percent        Cum.
------------+-----------------------------------
          0 |        338       33.14       33.14
          1 |        682       66.86      100.00
------------+-----------------------------------
      Total |      1,020      100.00
```

```
------------------------------------------------------------------------------------------------------------------------------------
-> quantile_hi = 4, treatment_binary_hi = 0

ChP_baselin |
          e |      Freq.     Percent        Cum.
------------+-----------------------------------
          0 |        142       29.52       29.52
          1 |        339       70.48      100.00
------------+-----------------------------------
      Total |        481      100.00

------------------------------------------------------------------------------------------------------------------------------------
-> quantile_hi = 4, treatment_binary_hi = 1

ChP_baselin |
          e |      Freq.     Percent        Cum.
------------+-----------------------------------
          0 |        317       30.19       30.19
          1 |        733       69.81      100.00
------------+-----------------------------------
      Total |      1,050      100.00

------------------------------------------------------------------------------------------------------------------------------------
-> quantile_hi = 5, treatment_binary_hi = 0

ChP_baselin |
          e |      Freq.     Percent        Cum.
------------+-----------------------------------
          0 |        162       35.76       35.76
          1 |        291       64.24      100.00
------------+-----------------------------------
      Total |        453      100.00

------------------------------------------------------------------------------------------------------------------------------------
-> quantile_hi = 5, treatment_binary_hi = 1

ChP_baselin |
          e |      Freq.     Percent        Cum.
------------+-----------------------------------
          0 |        379       35.59       35.59
          1 |        686       64.41      100.00
------------+-----------------------------------
      Total |      1,065      100.00


. by quantile_hi treatment_binary_hi, sort: summarize _06_age _11_init_pain_score treatment_hat_hi

------------------------------------------------------------------------------------------------------------------------------------
-> quantile_hi = 1, treatment_binary_hi = 0

    Variable |        Obs        Mean    Std. dev.       Min        Max
-------------+---------------------------------------------------------
     _06_age |        649    27.83205    6.228753         18         44
_11_init_p~e |        649    5.912173    1.837535          2         10
treatme~t_hi |        649    .5807995    .0180874   .5100691   .6058134

------------------------------------------------------------------------------------------------------------------------------------
-> quantile_hi = 1, treatment_binary_hi = 1

    Variable |        Obs        Mean    Std. dev.       Min        Max
-------------+---------------------------------------------------------
     _06_age |        880      27.725     6.27303         18         45
_11_init_p~e |        880    5.935227    1.772405          1         10
treatme~t_hi |        880    .5803038    .0180789   .5172513   .6058134

------------------------------------------------------------------------------------------------------------------------------------
-> quantile_hi = 2, treatment_binary_hi = 0
```

Variable	Obs	Mean	Std. dev.	Min	Max
_06_age	596	38.36577	5.777421	27	53
_11_init_p~e	596	5.540268	1.81035	1	10
treatme~t_hi	596	.6210038	.0092143	.6058758	.6370836

-> quantile_hi = 2, treatment_binary_hi = 1

Variable	Obs	Mean	Std. dev.	Min	Max
_06_age	929	38.324	5.836076	27	54
_11_init_p~e	929	5.517761	1.826516	1	10
treatme~t_hi	929	.6216189	.0089381	.605841	.6370836

-> quantile_hi = 3, treatment_binary_hi = 0

Variable	Obs	Mean	Std. dev.	Min	Max
_06_age	505	47.58614	5.841237	36	61
_11_init_p~e	505	5.50495	1.725301	1	10
treatme~t_hi	505	.650987	.0076572	.6370959	.6642949

-> quantile_hi = 3, treatment_binary_hi = 1

Variable	Obs	Mean	Std. dev.	Min	Max
_06_age	1,020	47.36863	5.912296	35	63
_11_init_p~e	1,020	5.497059	1.831196	1	10
treatme~t_hi	1,020	.6507318	.0076292	.6371226	.6642949

-> quantile_hi = 4, treatment_binary_hi = 0

Variable	Obs	Mean	Std. dev.	Min	Max
_06_age	481	54.97297	4.775949	43	68
_11_init_p~e	481	5.424116	1.637758	1	10
treatme~t_hi	481	.6772785	.0075312	.6643696	.6905739

-> quantile_hi = 4, treatment_binary_hi = 1

Variable	Obs	Mean	Std. dev.	Min	Max
_06_age	1,050	55.05905	4.93488	42	70
_11_init_p~e	1,050	5.426667	1.725393	1	10
treatme~t_hi	1,050	.6775119	.0076344	.664406	.6905739

-> quantile_hi = 5, treatment_binary_hi = 0

Variable	Obs	Mean	Std. dev.	Min	Max
_06_age	453	63.0883	6.194214	53	90
_11_init_p~e	453	4.962472	1.672501	1	10
treatme~t_hi	453	.7095527	.0157213	.6907577	.7621484

-> quantile_hi = 5, treatment_binary_hi = 1

Variable	Obs	Mean	Std. dev.	Min	Max
_06_age	1,065	63.8216	6.290142	51	87

```
_11_init_p~e |      1,065    4.902347    1.739048          1         10
treatme~t_hi |      1,065    .7114657     .016725   .6906803   .7861035


.
. by quantile_ii treatment_binary_ii, sort: tabulate female

-------------------------------------------------------------------------------------------------------------------------------
-> quantile_ii = 1, treatment_binary_ii = 0

     female |      Freq.     Percent        Cum.
------------+-----------------------------------
          0 |        343       26.43       26.43
          1 |        955       73.57      100.00
------------+-----------------------------------
      Total |      1,298      100.00

-------------------------------------------------------------------------------------------------------------------------------
-> quantile_ii = 1, treatment_binary_ii = 1

     female |      Freq.     Percent        Cum.
------------+-----------------------------------
          0 |         70       30.70       30.70
          1 |        158       69.30      100.00
------------+-----------------------------------
      Total |        228      100.00

-------------------------------------------------------------------------------------------------------------------------------
-> quantile_ii = 2, treatment_binary_ii = 0

     female |      Freq.     Percent        Cum.
------------+-----------------------------------
          0 |        334       26.11       26.11
          1 |        945       73.89      100.00
------------+-----------------------------------
      Total |      1,279      100.00

-------------------------------------------------------------------------------------------------------------------------------
-> quantile_ii = 2, treatment_binary_ii = 1

     female |      Freq.     Percent        Cum.
------------+-----------------------------------
          0 |         62       25.00       25.00
          1 |        186       75.00      100.00
------------+-----------------------------------
      Total |        248      100.00

-------------------------------------------------------------------------------------------------------------------------------
-> quantile_ii = 3, treatment_binary_ii = 0

     female |      Freq.     Percent        Cum.
------------+-----------------------------------
          0 |        390       30.30       30.30
          1 |        897       69.70      100.00
------------+-----------------------------------
      Total |      1,287      100.00

-------------------------------------------------------------------------------------------------------------------------------
-> quantile_ii = 3, treatment_binary_ii = 1

     female |      Freq.     Percent        Cum.
------------+-----------------------------------
          0 |         74       30.83       30.83
          1 |        166       69.17      100.00
------------+-----------------------------------
      Total |        240      100.00
```

-> quantile_ii = 4, treatment_binary_ii = 0

female	Freq.	Percent	Cum.
0	360	29.95	29.95
1	842	70.05	100.00
Total	1,202	100.00	

-> quantile_ii = 4, treatment_binary_ii = 1

female	Freq.	Percent	Cum.
0	83	25.86	25.86
1	238	74.14	100.00
Total	321	100.00	

-> quantile_ii = 5, treatment_binary_ii = 0

female	Freq.	Percent	Cum.
0	392	32.56	32.56
1	812	67.44	100.00
Total	1,204	100.00	

-> quantile_ii = 5, treatment_binary_ii = 1

female	Freq.	Percent	Cum.
0	109	33.96	33.96
1	212	66.04	100.00
Total	321	100.00	

. by quantile_ii treatment_binary_ii, sort: tabulate CPT_baseline

-> quantile_ii = 1, treatment_binary_ii = 0

CPT_baseline	Freq.	Percent	Cum.
0	707	54.47	54.47
1	591	45.53	100.00
Total	1,298	100.00	

-> quantile_ii = 1, treatment_binary_ii = 1

CPT_baseline	Freq.	Percent	Cum.
0	124	54.39	54.39
1	104	45.61	100.00
Total	228	100.00	

-> quantile_ii = 2, treatment_binary_ii = 0

```
CPT_baselin |
          e |      Freq.     Percent        Cum.
------------+-----------------------------------
          0 |        838       65.52       65.52
          1 |        441       34.48      100.00
------------+-----------------------------------
      Total |      1,279      100.00

------------------------------------------------------------------------------------------------------------------------
-> quantile_ii = 2, treatment_binary_ii = 1

CPT_baselin |
          e |      Freq.     Percent        Cum.
------------+-----------------------------------
          0 |        154       62.10       62.10
          1 |         94       37.90      100.00
------------+-----------------------------------
      Total |        248      100.00

------------------------------------------------------------------------------------------------------------------------
-> quantile_ii = 3, treatment_binary_ii = 0

CPT_baselin |
          e |      Freq.     Percent        Cum.
------------+-----------------------------------
          0 |        848       65.89       65.89
          1 |        439       34.11      100.00
------------+-----------------------------------
      Total |      1,287      100.00

------------------------------------------------------------------------------------------------------------------------
-> quantile_ii = 3, treatment_binary_ii = 1

CPT_baselin |
          e |      Freq.     Percent        Cum.
------------+-----------------------------------
          0 |        159       66.25       66.25
          1 |         81       33.75      100.00
------------+-----------------------------------
      Total |        240      100.00

------------------------------------------------------------------------------------------------------------------------
-> quantile_ii = 4, treatment_binary_ii = 0

CPT_baselin |
          e |      Freq.     Percent        Cum.
------------+-----------------------------------
          0 |        866       72.05       72.05
          1 |        336       27.95      100.00
------------+-----------------------------------
      Total |      1,202      100.00

------------------------------------------------------------------------------------------------------------------------
-> quantile_ii = 4, treatment_binary_ii = 1

CPT_baselin |
          e |      Freq.     Percent        Cum.
------------+-----------------------------------
          0 |        232       72.27       72.27
          1 |         89       27.73      100.00
------------+-----------------------------------
      Total |        321      100.00

------------------------------------------------------------------------------------------------------------------------
-> quantile_ii = 5, treatment_binary_ii = 0
```

```
CPT_baselin |
          e |      Freq.     Percent        Cum.
------------+-----------------------------------
          0 |        953       79.15       79.15
          1 |        251       20.85      100.00
------------+-----------------------------------
      Total |      1,204      100.00

-------------------------------------------------------------------------------------------------------------------------------
-> quantile_ii = 5, treatment_binary_ii = 1

CPT_baselin |
          e |      Freq.     Percent        Cum.
------------+-----------------------------------
          0 |        263       81.93       81.93
          1 |         58       18.07      100.00
------------+-----------------------------------
      Total |        321      100.00


. by quantile_ii treatment_binary_ii, sort: tabulate CPM_baseline

-------------------------------------------------------------------------------------------------------------------------------
-> quantile_ii = 1, treatment_binary_ii = 0

CPM_baselin |
          e |      Freq.     Percent        Cum.
------------+-----------------------------------
          0 |      1,194       91.99       91.99
          1 |        104        8.01      100.00
------------+-----------------------------------
      Total |      1,298      100.00

-------------------------------------------------------------------------------------------------------------------------------
-> quantile_ii = 1, treatment_binary_ii = 1

CPM_baselin |
          e |      Freq.     Percent        Cum.
------------+-----------------------------------
          0 |        215       94.30       94.30
          1 |         13        5.70      100.00
------------+-----------------------------------
      Total |        228      100.00

-------------------------------------------------------------------------------------------------------------------------------
-> quantile_ii = 2, treatment_binary_ii = 0

CPM_baselin |
          e |      Freq.     Percent        Cum.
------------+-----------------------------------
          0 |        989       77.33       77.33
          1 |        290       22.67      100.00
------------+-----------------------------------
      Total |      1,279      100.00

-------------------------------------------------------------------------------------------------------------------------------
-> quantile_ii = 2, treatment_binary_ii = 1

CPM_baselin |
          e |      Freq.     Percent        Cum.
------------+-----------------------------------
          0 |        197       79.44       79.44
          1 |         51       20.56      100.00
------------+-----------------------------------
      Total |        248      100.00

-------------------------------------------------------------------------------------------------------------------------------
```

```
-> quantile_ii = 3, treatment_binary_ii = 0

CPM_baselin |
          e |      Freq.     Percent        Cum.
------------+-----------------------------------
          0 |        822       63.87       63.87
          1 |        465       36.13      100.00
------------+-----------------------------------
      Total |      1,287      100.00

-----------------------------------------------------------------------------------------------------------------------------------
-> quantile_ii = 3, treatment_binary_ii = 1

CPM_baselin |
          e |      Freq.     Percent        Cum.
------------+-----------------------------------
          0 |        144       60.00       60.00
          1 |         96       40.00      100.00
------------+-----------------------------------
      Total |        240      100.00

-----------------------------------------------------------------------------------------------------------------------------------
-> quantile_ii = 4, treatment_binary_ii = 0

CPM_baselin |
          e |      Freq.     Percent        Cum.
------------+-----------------------------------
          0 |        853       70.97       70.97
          1 |        349       29.03      100.00
------------+-----------------------------------
      Total |      1,202      100.00

-----------------------------------------------------------------------------------------------------------------------------------
-> quantile_ii = 4, treatment_binary_ii = 1

CPM_baselin |
          e |      Freq.     Percent        Cum.
------------+-----------------------------------
          0 |        231       71.96       71.96
          1 |         90       28.04      100.00
------------+-----------------------------------
      Total |        321      100.00

-----------------------------------------------------------------------------------------------------------------------------------
-> quantile_ii = 5, treatment_binary_ii = 0

CPM_baselin |
          e |      Freq.     Percent        Cum.
------------+-----------------------------------
          0 |        759       63.04       63.04
          1 |        445       36.96      100.00
------------+-----------------------------------
      Total |      1,204      100.00

-----------------------------------------------------------------------------------------------------------------------------------
-> quantile_ii = 5, treatment_binary_ii = 1

CPM_baselin |
          e |      Freq.     Percent        Cum.
------------+-----------------------------------
          0 |        190       59.19       59.19
          1 |        131       40.81      100.00
------------+-----------------------------------
      Total |        321      100.00


. by quantile_ii treatment_binary_ii, sort: tabulate ChP_baseline
```

```
-----------------------------------------------------------------------------------------------------------------------------
-> quantile_ii = 1, treatment_binary_ii = 0

ChP_baselin |
          e |      Freq.     Percent        Cum.
------------+-----------------------------------
          0 |        387       29.82       29.82
          1 |        911       70.18      100.00
------------+-----------------------------------
      Total |      1,298      100.00

-----------------------------------------------------------------------------------------------------------------------------
-> quantile_ii = 1, treatment_binary_ii = 1

ChP_baselin |
          e |      Freq.     Percent        Cum.
------------+-----------------------------------
          0 |         78       34.21       34.21
          1 |        150       65.79      100.00
------------+-----------------------------------
      Total |        228      100.00

-----------------------------------------------------------------------------------------------------------------------------
-> quantile_ii = 2, treatment_binary_ii = 0

ChP_baselin |
          e |      Freq.     Percent        Cum.
------------+-----------------------------------
          0 |        394       30.81       30.81
          1 |        885       69.19      100.00
------------+-----------------------------------
      Total |      1,279      100.00

-----------------------------------------------------------------------------------------------------------------------------
-> quantile_ii = 2, treatment_binary_ii = 1

ChP_baselin |
          e |      Freq.     Percent        Cum.
------------+-----------------------------------
          0 |         81       32.66       32.66
          1 |        167       67.34      100.00
------------+-----------------------------------
      Total |        248      100.00

-----------------------------------------------------------------------------------------------------------------------------
-> quantile_ii = 3, treatment_binary_ii = 0

ChP_baselin |
          e |      Freq.     Percent        Cum.
------------+-----------------------------------
          0 |        402       31.24       31.24
          1 |        885       68.76      100.00
------------+-----------------------------------
      Total |      1,287      100.00

-----------------------------------------------------------------------------------------------------------------------------
-> quantile_ii = 3, treatment_binary_ii = 1

ChP_baselin |
          e |      Freq.     Percent        Cum.
------------+-----------------------------------
          0 |         62       25.83       25.83
          1 |        178       74.17      100.00
------------+-----------------------------------
      Total |        240      100.00
```

```
-----------------------------------------------------------------------------------------------------------------------------------------
-> quantile_ii = 4, treatment_binary_ii = 0

ChP_baselin |
          e |      Freq.     Percent        Cum.
------------+-----------------------------------
          0 |        409       34.03       34.03
          1 |        793       65.97      100.00
------------+-----------------------------------
      Total |      1,202      100.00

-----------------------------------------------------------------------------------------------------------------------------------------
-> quantile_ii = 4, treatment_binary_ii = 1

ChP_baselin |
          e |      Freq.     Percent        Cum.
------------+-----------------------------------
          0 |        108       33.64       33.64
          1 |        213       66.36      100.00
------------+-----------------------------------
      Total |        321      100.00

-----------------------------------------------------------------------------------------------------------------------------------------
-> quantile_ii = 5, treatment_binary_ii = 0

ChP_baselin |
          e |      Freq.     Percent        Cum.
------------+-----------------------------------
          0 |        434       36.05       36.05
          1 |        770       63.95      100.00
------------+-----------------------------------
      Total |      1,204      100.00

-----------------------------------------------------------------------------------------------------------------------------------------
-> quantile_ii = 5, treatment_binary_ii = 1

ChP_baselin |
          e |      Freq.     Percent        Cum.
------------+-----------------------------------
          0 |        114       35.51       35.51
          1 |        207       64.49      100.00
------------+-----------------------------------
      Total |        321      100.00


. by quantile_ii treatment_binary_ii, sort: summarize _06_age _11_init_pain_score treatment_hat_ii

-----------------------------------------------------------------------------------------------------------------------------------------
-> quantile_ii = 1, treatment_binary_ii = 0

    Variable |        Obs        Mean    Std. dev.       Min        Max
-------------+---------------------------------------------------------
     _06_age |      1,298    63.09861    6.564864         50         90
_11_init_p~e |      1,298    4.948382    1.688444          1         10
treatme~t_ii |      1,298    .1462897    .0076393   .1103216   .1558711

-----------------------------------------------------------------------------------------------------------------------------------------
-> quantile_ii = 1, treatment_binary_ii = 1

    Variable |        Obs        Mean    Std. dev.       Min        Max
-------------+---------------------------------------------------------
     _06_age |        228    62.57895    6.348741         50         82
_11_init_p~e |        228    5.096491    1.699802          1         10
treatme~t_ii |        228    .1468731    .0072443   .1196765   .1558195

-----------------------------------------------------------------------------------------------------------------------------------------
-> quantile_ii = 2, treatment_binary_ii = 0
```

Variable	Obs	Mean	Std. dev.	Min	Max
_06_age	1,279	54.44253	5.987044	41	72
_11_init_p~e	1,279	5.435496	1.759932	1	10
treatme~t_ii	1,279	.1630958	.0039626	.1559074	.1697194

-> quantile_ii = 2, treatment_binary_ii = 1

Variable	Obs	Mean	Std. dev.	Min	Max
_06_age	248	53.92339	5.302029	42	70
_11_init_p~e	248	5.370968	1.532206	1	9
treatme~t_ii	248	.1631158	.0040437	.1561	.1696943

-> quantile_ii = 3, treatment_binary_ii = 0

Variable	Obs	Mean	Std. dev.	Min	Max
_06_age	1,287	47.63559	7.180417	32	65
_11_init_p~e	1,287	5.407148	1.827017	1	10
treatme~t_ii	1,287	.1764735	.0038452	.1697345	.1832667

-> quantile_ii = 3, treatment_binary_ii = 1

Variable	Obs	Mean	Std. dev.	Min	Max
_06_age	240	48.15417	7.055457	34	63
_11_init_p~e	240	5.4375	1.598983	1	10
treatme~t_ii	240	.1764539	.0038542	.1699227	.1832667

-> quantile_ii = 4, treatment_binary_ii = 0

Variable	Obs	Mean	Std. dev.	Min	Max
_06_age	1,202	38.56073	7.120941	23	57
_11_init_p~e	1,202	5.580699	1.843293	1	10
treatme~t_ii	1,202	.1912169	.0048071	.1832808	.1997181

-> quantile_ii = 4, treatment_binary_ii = 1

Variable	Obs	Mean	Std. dev.	Min	Max
_06_age	321	38.46729	6.850617	25	55
_11_init_p~e	321	5.626168	1.781099	1	10
treatme~t_ii	321	.1912333	.0048254	.183349	.1997181

-> quantile_ii = 5, treatment_binary_ii = 0

Variable	Obs	Mean	Std. dev.	Min	Max
_06_age	1,204	28.3289	6.949273	18	48
_11_init_p~e	1,204	5.925249	1.824665	1	10
treatme~t_ii	1,204	.2130015	.0103638	.1997283	.2551354

-> quantile_ii = 5, treatment_binary_ii = 1

Variable	Obs	Mean	Std. dev.	Min	Max
_06_age	321	28.76947	7.220834	18	46

```
_11_init_p~e |        321    5.825545    1.757333          2         10
treatme~t_ii |        321    .2131587    .0109136   .1997283   .2574059


.
. by quantile_li treatment_binary_li, sort: tabulate female

-------------------------------------------------------------------------------------------------------------------------------------
-> quantile_li = 1, treatment_binary_li = 0

     female |      Freq.     Percent        Cum.
------------+-----------------------------------
          0 |        502       37.77       37.77
          1 |        827       62.23      100.00
------------+-----------------------------------
      Total |      1,329      100.00

-------------------------------------------------------------------------------------------------------------------------------------
-> quantile_li = 1, treatment_binary_li = 1

     female |      Freq.     Percent        Cum.
------------+-----------------------------------
          0 |         73       36.87       36.87
          1 |        125       63.13      100.00
------------+-----------------------------------
      Total |        198      100.00

-------------------------------------------------------------------------------------------------------------------------------------
-> quantile_li = 2, treatment_binary_li = 0

     female |      Freq.     Percent        Cum.
------------+-----------------------------------
          0 |        319       24.22       24.22
          1 |        998       75.78      100.00
------------+-----------------------------------
      Total |      1,317      100.00

-------------------------------------------------------------------------------------------------------------------------------------
-> quantile_li = 2, treatment_binary_li = 1

     female |      Freq.     Percent        Cum.
------------+-----------------------------------
          0 |         57       26.39       26.39
          1 |        159       73.61      100.00
------------+-----------------------------------
      Total |        216      100.00

-------------------------------------------------------------------------------------------------------------------------------------
-> quantile_li = 3, treatment_binary_li = 0

     female |      Freq.     Percent        Cum.
------------+-----------------------------------
          0 |        371       29.24       29.24
          1 |        898       70.76      100.00
------------+-----------------------------------
      Total |      1,269      100.00

-------------------------------------------------------------------------------------------------------------------------------------
-> quantile_li = 3, treatment_binary_li = 1

     female |      Freq.     Percent        Cum.
------------+-----------------------------------
          0 |         72       29.03       29.03
          1 |        176       70.97      100.00
------------+-----------------------------------
      Total |        248      100.00
```

```
----------------------------------------------------------------------------------------------------------------------------------
-> quantile_li = 4, treatment_binary_li = 0

     female |      Freq.     Percent        Cum.
------------+-----------------------------------
          0 |        390       30.61       30.61
          1 |        884       69.39      100.00
------------+-----------------------------------
      Total |      1,274      100.00

----------------------------------------------------------------------------------------------------------------------------------
-> quantile_li = 4, treatment_binary_li = 1

     female |      Freq.     Percent        Cum.
------------+-----------------------------------
          0 |         62       24.60       24.60
          1 |        190       75.40      100.00
------------+-----------------------------------
      Total |        252      100.00

----------------------------------------------------------------------------------------------------------------------------------
-> quantile_li = 5, treatment_binary_li = 0

     female |      Freq.     Percent        Cum.
------------+-----------------------------------
          0 |        288       23.61       23.61
          1 |        932       76.39      100.00
------------+-----------------------------------
      Total |      1,220      100.00

----------------------------------------------------------------------------------------------------------------------------------
-> quantile_li = 5, treatment_binary_li = 1

     female |      Freq.     Percent        Cum.
------------+-----------------------------------
          0 |         83       27.21       27.21
          1 |        222       72.79      100.00
------------+-----------------------------------
      Total |        305      100.00


. by quantile_li treatment_binary_li, sort: tabulate CPT_baseline

----------------------------------------------------------------------------------------------------------------------------------
-> quantile_li = 1, treatment_binary_li = 0

CPT_baselin |
          e |      Freq.     Percent        Cum.
------------+-----------------------------------
          0 |      1,086       81.72       81.72
          1 |        243       18.28      100.00
------------+-----------------------------------
      Total |      1,329      100.00

----------------------------------------------------------------------------------------------------------------------------------
-> quantile_li = 1, treatment_binary_li = 1

CPT_baselin |
          e |      Freq.     Percent        Cum.
------------+-----------------------------------
          0 |        172       86.87       86.87
          1 |         26       13.13      100.00
------------+-----------------------------------
      Total |        198      100.00

----------------------------------------------------------------------------------------------------------------------------------
-> quantile_li = 2, treatment_binary_li = 0
```

CPT_baseline	Freq.	Percent	Cum.
0	884	67.12	67.12
1	433	32.88	100.00
Total	1,317	100.00	

-> quantile_li = 2, treatment_binary_li = 1

CPT_baseline	Freq.	Percent	Cum.
0	137	63.43	63.43
1	79	36.57	100.00
Total	216	100.00	

-> quantile_li = 3, treatment_binary_li = 0

CPT_baseline	Freq.	Percent	Cum.
0	809	63.75	63.75
1	460	36.25	100.00
Total	1,269	100.00	

-> quantile_li = 3, treatment_binary_li = 1

CPT_baseline	Freq.	Percent	Cum.
0	154	62.10	62.10
1	94	37.90	100.00
Total	248	100.00	

-> quantile_li = 4, treatment_binary_li = 0

CPT_baseline	Freq.	Percent	Cum.
0	830	65.15	65.15
1	444	34.85	100.00
Total	1,274	100.00	

-> quantile_li = 4, treatment_binary_li = 1

CPT_baseline	Freq.	Percent	Cum.
0	175	69.44	69.44
1	77	30.56	100.00
Total	252	100.00	

-> quantile_li = 5, treatment_binary_li = 0

```
CPT_baselin |
          e |      Freq.     Percent        Cum.
------------+-----------------------------------
          0 |        726       59.51       59.51
          1 |        494       40.49      100.00
------------+-----------------------------------
      Total |      1,220      100.00

--------------------------------------------------------------------------------------------------------------------------------
-> quantile_li = 5, treatment_binary_li = 1

CPT_baselin |
          e |      Freq.     Percent        Cum.
------------+-----------------------------------
          0 |        171       56.07       56.07
          1 |        134       43.93      100.00
------------+-----------------------------------
      Total |        305      100.00


. by quantile_li treatment_binary_li, sort: tabulate CPM_baseline

--------------------------------------------------------------------------------------------------------------------------------
-> quantile_li = 1, treatment_binary_li = 0

CPM_baselin |
          e |      Freq.     Percent        Cum.
------------+-----------------------------------
          0 |      1,027       77.28       77.28
          1 |        302       22.72      100.00
------------+-----------------------------------
      Total |      1,329      100.00

--------------------------------------------------------------------------------------------------------------------------------
-> quantile_li = 1, treatment_binary_li = 1

CPM_baselin |
          e |      Freq.     Percent        Cum.
------------+-----------------------------------
          0 |        144       72.73       72.73
          1 |         54       27.27      100.00
------------+-----------------------------------
      Total |        198      100.00

--------------------------------------------------------------------------------------------------------------------------------
-> quantile_li = 2, treatment_binary_li = 0

CPM_baselin |
          e |      Freq.     Percent        Cum.
------------+-----------------------------------
          0 |        929       70.54       70.54
          1 |        388       29.46      100.00
------------+-----------------------------------
      Total |      1,317      100.00

--------------------------------------------------------------------------------------------------------------------------------
-> quantile_li = 2, treatment_binary_li = 1

CPM_baselin |
          e |      Freq.     Percent        Cum.
------------+-----------------------------------
          0 |        158       73.15       73.15
          1 |         58       26.85      100.00
------------+-----------------------------------
      Total |        216      100.00

--------------------------------------------------------------------------------------------------------------------------------
```

```
-> quantile_li = 3, treatment_binary_li = 0

CPM_baselin |
          e |      Freq.     Percent        Cum.
------------+-----------------------------------
          0 |        855       67.38       67.38
          1 |        414       32.62      100.00
------------+-----------------------------------
      Total |      1,269      100.00

-------------------------------------------------------------------------------------------------------------------------------
-> quantile_li = 3, treatment_binary_li = 1

CPM_baselin |
          e |      Freq.     Percent        Cum.
------------+-----------------------------------
          0 |        172       69.35       69.35
          1 |         76       30.65      100.00
------------+-----------------------------------
      Total |        248      100.00

-------------------------------------------------------------------------------------------------------------------------------
-> quantile_li = 4, treatment_binary_li = 0

CPM_baselin |
          e |      Freq.     Percent        Cum.
------------+-----------------------------------
          0 |        953       74.80       74.80
          1 |        321       25.20      100.00
------------+-----------------------------------
      Total |      1,274      100.00

-------------------------------------------------------------------------------------------------------------------------------
-> quantile_li = 4, treatment_binary_li = 1

CPM_baselin |
          e |      Freq.     Percent        Cum.
------------+-----------------------------------
          0 |        184       73.02       73.02
          1 |         68       26.98      100.00
------------+-----------------------------------
      Total |        252      100.00

-------------------------------------------------------------------------------------------------------------------------------
-> quantile_li = 5, treatment_binary_li = 0

CPM_baselin |
          e |      Freq.     Percent        Cum.
------------+-----------------------------------
          0 |        935       76.64       76.64
          1 |        285       23.36      100.00
------------+-----------------------------------
      Total |      1,220      100.00

-------------------------------------------------------------------------------------------------------------------------------
-> quantile_li = 5, treatment_binary_li = 1

CPM_baselin |
          e |      Freq.     Percent        Cum.
------------+-----------------------------------
          0 |        237       77.70       77.70
          1 |         68       22.30      100.00
------------+-----------------------------------
      Total |        305      100.00


. by quantile_li treatment_binary_li, sort: tabulate ChP_baseline
```

```
----------------------------------------------------------------------------------------------------------------------------------
-> quantile_li = 1, treatment_binary_li = 0

ChP_baselin |
          e |      Freq.     Percent        Cum.
------------+-----------------------------------
          0 |        524       39.43       39.43
          1 |        805       60.57      100.00
------------+-----------------------------------
      Total |      1,329      100.00

----------------------------------------------------------------------------------------------------------------------------------
-> quantile_li = 1, treatment_binary_li = 1

ChP_baselin |
          e |      Freq.     Percent        Cum.
------------+-----------------------------------
          0 |         72       36.36       36.36
          1 |        126       63.64      100.00
------------+-----------------------------------
      Total |        198      100.00

----------------------------------------------------------------------------------------------------------------------------------
-> quantile_li = 2, treatment_binary_li = 0

ChP_baselin |
          e |      Freq.     Percent        Cum.
------------+-----------------------------------
          0 |        406       30.83       30.83
          1 |        911       69.17      100.00
------------+-----------------------------------
      Total |      1,317      100.00

----------------------------------------------------------------------------------------------------------------------------------
-> quantile_li = 2, treatment_binary_li = 1

ChP_baselin |
          e |      Freq.     Percent        Cum.
------------+-----------------------------------
          0 |         58       26.85       26.85
          1 |        158       73.15      100.00
------------+-----------------------------------
      Total |        216      100.00

----------------------------------------------------------------------------------------------------------------------------------
-> quantile_li = 3, treatment_binary_li = 0

ChP_baselin |
          e |      Freq.     Percent        Cum.
------------+-----------------------------------
          0 |        412       32.47       32.47
          1 |        857       67.53      100.00
------------+-----------------------------------
      Total |      1,269      100.00

----------------------------------------------------------------------------------------------------------------------------------
-> quantile_li = 3, treatment_binary_li = 1

ChP_baselin |
          e |      Freq.     Percent        Cum.
------------+-----------------------------------
          0 |         76       30.65       30.65
          1 |        172       69.35      100.00
------------+-----------------------------------
      Total |        248      100.00
```

```
------------------------------------------------------------------------------------------------------------------------
-> quantile_li = 4, treatment_binary_li = 0

ChP_baselin |
          e |      Freq.     Percent        Cum.
------------+-----------------------------------
          0 |        431       33.83       33.83
          1 |        843       66.17      100.00
------------+-----------------------------------
      Total |      1,274      100.00

------------------------------------------------------------------------------------------------------------------------
-> quantile_li = 4, treatment_binary_li = 1

ChP_baselin |
          e |      Freq.     Percent        Cum.
------------+-----------------------------------
          0 |         82       32.54       32.54
          1 |        170       67.46      100.00
------------+-----------------------------------
      Total |        252      100.00

------------------------------------------------------------------------------------------------------------------------
-> quantile_li = 5, treatment_binary_li = 0

ChP_baselin |
          e |      Freq.     Percent        Cum.
------------+-----------------------------------
          0 |        308       25.25       25.25
          1 |        912       74.75      100.00
------------+-----------------------------------
      Total |      1,220      100.00

------------------------------------------------------------------------------------------------------------------------
-> quantile_li = 5, treatment_binary_li = 1

ChP_baselin |
          e |      Freq.     Percent        Cum.
------------+-----------------------------------
          0 |        100       32.79       32.79
          1 |        205       67.21      100.00
------------+-----------------------------------
      Total |        305      100.00


. by quantile_li treatment_binary_li, sort: summarize _06_age _11_init_pain_score treatment_hat_li

------------------------------------------------------------------------------------------------------------------------
-> quantile_li = 1, treatment_binary_li = 0

    Variable |        Obs        Mean    Std. dev.       Min        Max
-------------+---------------------------------------------------------
     _06_age |      1,329    63.98796    6.150828         52         87
_11_init_p~e |      1,329    5.360421    1.803268          1         10
treatme~t_li |      1,329    .1291872     .007649   .0972563   .1387815

------------------------------------------------------------------------------------------------------------------------
-> quantile_li = 1, treatment_binary_li = 1

    Variable |        Obs        Mean    Std. dev.       Min        Max
-------------+---------------------------------------------------------
     _06_age |        198    63.50505    5.995555         52         90
_11_init_p~e |        198    5.373737     1.65922          2         10
treatme~t_li |        198    .1298885    .0071788   .1073444   .1387815

------------------------------------------------------------------------------------------------------------------------
-> quantile_li = 2, treatment_binary_li = 0
```

Variable	Obs	Mean	Std. dev.	Min	Max
_06_age	1,317	55.08656	4.212275	44	67
_11_init_p~e	1,317	5.494305	1.730999	1	10
treatme~t_li	1,317	.1449562	.0034388	.1388047	.1508268

-> quantile_li = 2, treatment_binary_li = 1

Variable	Obs	Mean	Std. dev.	Min	Max
_06_age	216	55.36574	4.13949	44	63
_11_init_p~e	216	5.481481	1.80944	1	10
treatme~t_li	216	.1447938	.0036209	.1389287	.1508268

-> quantile_li = 3, treatment_binary_li = 0

Variable	Obs	Mean	Std. dev.	Min	Max
_06_age	1,269	47.38061	5.241875	35	60
_11_init_p~e	1,269	5.51379	1.789606	1	10
treatme~t_li	1,269	.157621	.0039921	.1508433	.1646879

-> quantile_li = 3, treatment_binary_li = 1

Variable	Obs	Mean	Std. dev.	Min	Max
_06_age	248	47.35081	5.168607	36	60
_11_init_p~e	248	5.41129	1.817647	1	10
treatme~t_li	248	.1578156	.0036752	.1509002	.1644891

-> quantile_li = 4, treatment_binary_li = 0

Variable	Obs	Mean	Std. dev.	Min	Max
_06_age	1,274	38.27237	5.05496	26	51
_11_init_p~e	1,274	5.419152	1.818594	1	10
treatme~t_li	1,274	.1725989	.0048173	.1647104	.1813426

-> quantile_li = 4, treatment_binary_li = 1

Variable	Obs	Mean	Std. dev.	Min	Max
_06_age	252	38.16667	5.023054	28	51
_11_init_p~e	252	5.626984	1.828217	1	10
treatme~t_li	252	.1728345	.0049148	.1647104	.1813138

-> quantile_li = 5, treatment_binary_li = 0

Variable	Obs	Mean	Std. dev.	Min	Max
_06_age	1,220	27.35902	5.643697	18	42
_11_init_p~e	1,220	5.513934	1.810271	1	10
treatme~t_li	1,220	.1946976	.0101548	.1814277	.2297473

-> quantile_li = 5, treatment_binary_li = 1

Variable	Obs	Mean	Std. dev.	Min	Max
_06_age	305	27.32459	5.609809	18	42

_11_init_p~e |	305	5.42623	1.926776	1	10
treatme~t_li |	305	.1943405	.0092391	.1814632	.2241988

.
end of do-file

.
