# Supplementary material for: The Effect of Therapeutic Adherence on the Effectiveness of a Digital Therapeutic Exercise Program: A Propensity Score Matching Analysis
Source: Healthcare (Basel). 2023 Sep 23;11(19):2614. doi: 10.3390/healthcare11192614 (PMC10572588; doi:10.3390/healthcare11192614)
Supplement: Supplementary file 1 [file healthcare-11-02614-s001.zip › Supplementary_2.pdf]

Supplementary File 2: Graphical assessment of a region of common support of propensity scores (PS) for patients with high-intensity (hi), intermediate-intensity (ii), and low-intensity (li) use profiles.

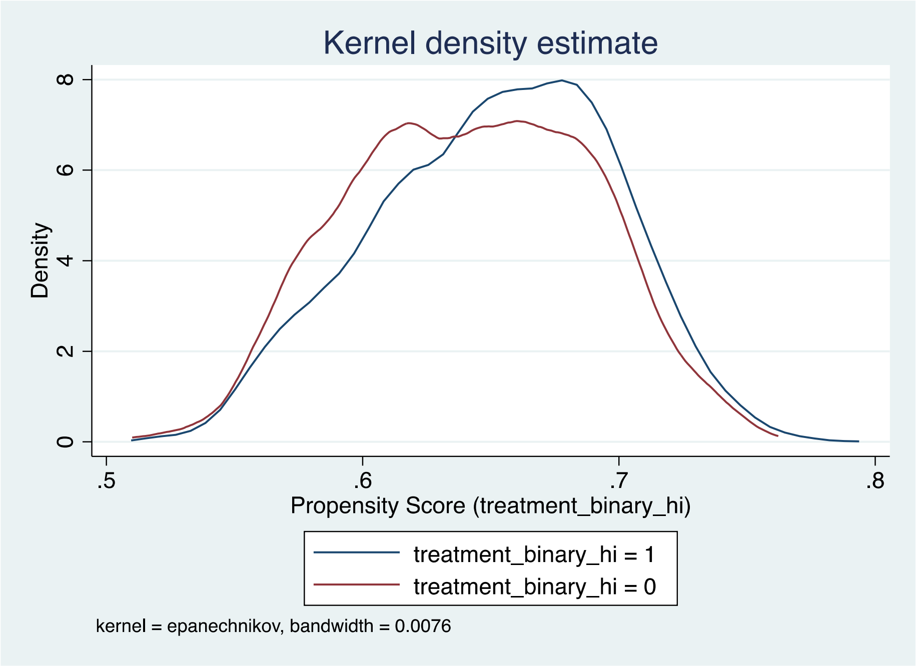

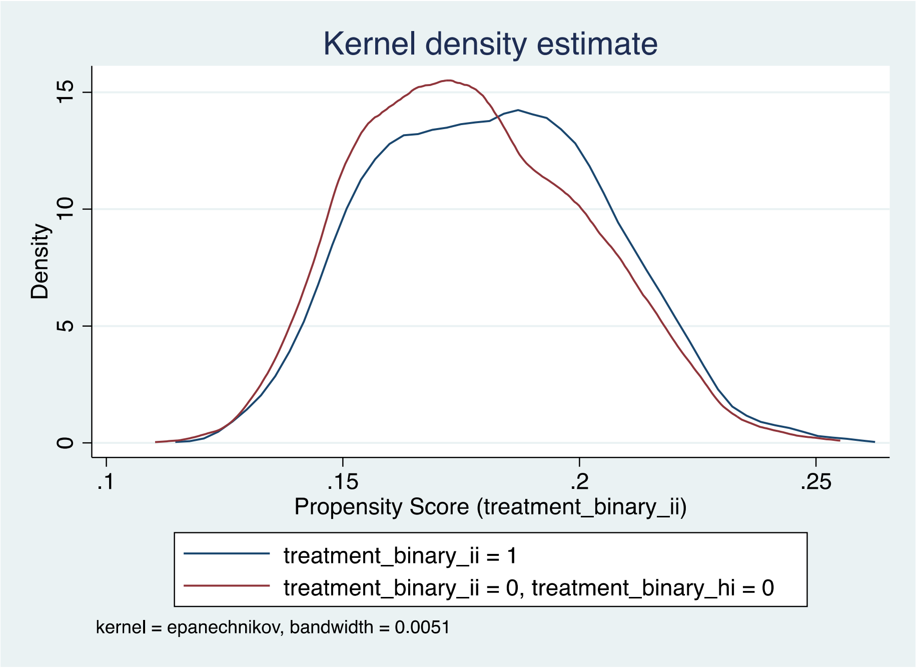

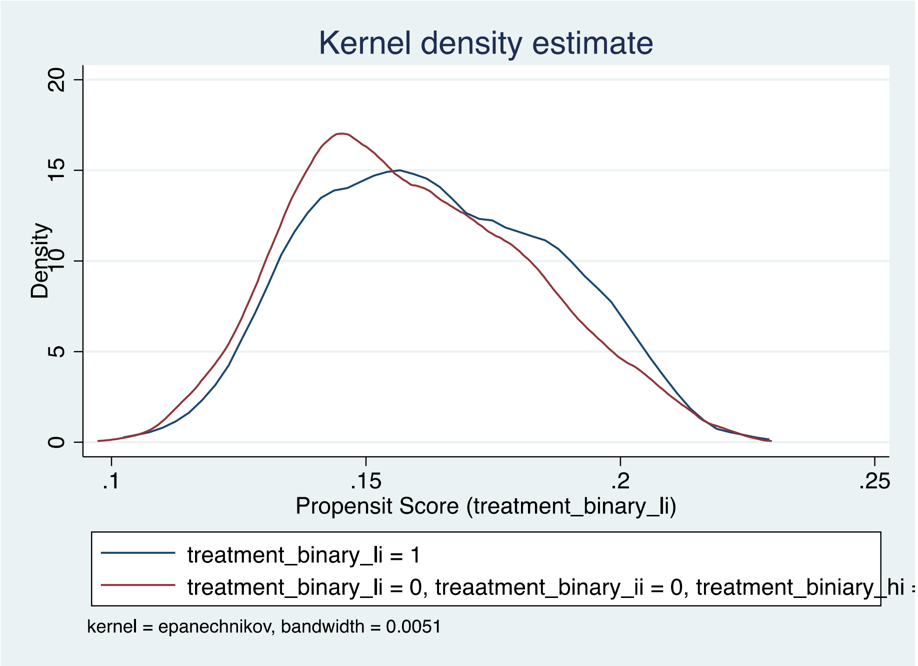
Kernel density estimate
Density
0
5
10
15
20
.1
.15
.2
.25
Propensit Score (treatment_binary_li)
treatment_binary_li = 1
treatment_binary_li = 0, treaatment_binary_ii = 0, treatment_biniary_hi =
kernel = epanechnikov, bandwidth = 0.0051
