# Supplementary material for: The Effect of Therapeutic Adherence on the Effectiveness of a Digital Therapeutic Exercise Program: A Propensity Score Matching Analysis
Source: Healthcare (Basel). 2023 Sep 23;11(19):2614. doi: 10.3390/healthcare11192614 (PMC10572588; doi:10.3390/healthcare11192614)
Supplement: Supplementary file 1 [file healthcare-11-02614-s001.zip › Supplementary_3.pdf]

Supplementary File 3: Full guidelines for reporting propensity score analysis, modified From the STROBE (STrengthening the Reporting of OBservational studies in Epidemiology) Statement*. ‘X’ in the item column indicates that the respective item has been addressed in the article, whereas ‘n/a’ indicates that the item is not applicable. Any number (eg, ‘1’) refers to the index below for a further explanation.

Section/topic	Item	No	Recommendation
**Title and abstract**	X	1	Indicate the use of propensity analysis with a commonly used term in the title or the abstract
	X	2	Provide in the abstract an informative and balanced summary of what was done and what was found
**Introduction**			
Background/rationale	X	3	Explain the scientific background and rationale for the investigation being reported
Objectives	X	4	State specific objectives, including any prespecified hypotheses
**Methods**			
Setting	X	5	Describe the setting, locations, and relevant dates, including periods of recruitment, treatment, follow-up, and data collection
Patient selection	X	6	Give the eligibility criteria, and the sources and methods of subject ascertainment and selection
Variables	X	7	Clearly define all outcomes, treatments, predictors. Give diagnostic criteria, if applicable
Data sources/ measurement	X	8	For each variable of interest, give sources of data and details of methods of assessment (measurement)
Bias	X	9	Describe how propensity score analysis was used to address bias
	X	10	Describe any other methods to address potential sources of bias, e.g. sensitivity analysis
Sample size	X	11	Explain how the study size was arrived at
Statistical analyses	X	12	Describe all the analytic methods, including the propensity score methods, e.g. matching, weighting, stratification, or covariate adjustment using propensity score
	X	13	Indicate the model used to estimate propensity score, e.g. logistic model, boosting (meta-classifiers), decision trees
	X	14	State the variables included in the propensity score model
	X	15	Explain the variable selection procedure for propensity score model
		16	For propensity score matching:
	X	16.1	Explicitly state the matching algorithm and distance metric
	X	16.2	Indicate matching ratio (1:m matching)
	X	16.3	Indicate whether sampling with or without replacement was used

	X	16.4	Describe the statistical methods for the analysis of matched data
	X	16.5	Describe methods for assessing the comparability of baseline characteristics in the matched groups
	n/a	17	For propensity score weighting, describe methods for assessing the comparability of baseline characteristics in the weighted groups
		18	For propensity score stratification:
	n/a	18.1	Give the number of strata
	n/a	18.2	Describe methods for assessing the comparability of baseline characteristics in each stratum
	X	19	Explain how assumption of propensity score analysis was examined
	X	20	Explain how missing data were addressed, including missing data in propensity score estimation
	n/a	21	If applicable, describe any methods used to examine subgroups and interactions
	X	22	Describe any sensitivity analyses
	X	23	Indicate the software used for analysis
	n/a	24	If applicable, report the package used to create matched sample, e.g. GMATCH macro in SAS, MatchIt package®, Optmatch package ®
**Results**			
Participants		25	Report numbers of participants at each stage of study:
	X	25.1	sample size of patients potentially eligible
	X	25.2	sample size of patients confirmed eligible and included
	X	25.3	sample size of patients analyzed
	X	25.4	for propensity score matching, sample size for each treatment group before and after matching
	n/a	26	Explain reasons for exclusion at each stage
	1	27	Consider use of a flow diagram
Patient characteristics	X	28	Describe the distribution of baseline characteristics for each group before propensity score analysis
		29	For propensity score matching, weighting, or stratification:
	X	29.1	Describe the distribution of baseline characteristics in the matched/weighted groups or in each stratum
	X	29.2	Describe the results of the comparability of baseline characteristics, whether there are still systematic differences between treatment groups
	X	30	Indicate number of patients with missing data for each variable of interest, especially the variables used in propensity score model
Outcome data	X	31	Report outcomes of each treatment group

Main results	X	32	Give propensity score analysis estimates and their precision, e.g. 95% confidence interval
	n/a	33	If applicable, give unadjusted estimates and/or adjusted estimates and their precision, e.g. 95% confidence interval. Make clear which additional factors were adjusted for
Other analyses	X	34	Report other analyses done, e.g. analyses of subgroups and interactions, and sensitivity analyses
**Discussion**			
Key results	X	35	Summarize key results with reference to study objectives
Limitations	X	36	Discuss limitations of the study, taking into account sources of potential bias or imprecision
	X	37	Discuss both direction and magnitude of any potential bias
Interpretation	X	38	Discuss whether imbalance of baseline characteristics still exists, and give a cautious interpretation
	X	39	Give a cautious overall interpretation of results considering objectives, limitations, multiplicity of analyses, results from similar studies, and other relevant evidence
Generalizability	X	40	For propensity score matching, discuss the possibility and potential influence of incomplete matching, especially the studies in which the matched sample size is less than 50%
**Other information**			
Funding	X	41	Give the source of funding and the role of the funders for the present study and, if applicable, for the original study on which the present article is based

* von Elm E, Altman DG, Egger M, et al. The Strengthening the Reporting of Observational Studies in Epidemiology (STROBE) statement: guidelines for reporting observational studies. J Clin Epidemiol 2008;61(4):344-9.

1: We did not provide a flow diagram, as all patients enrolled in the given period were included in this analysis. As the submission of the baseline variables was mandatory for the start of the exercise program, there was no exclusion based on incomplete data sets.
