# Supplementary material for: The Effect of Therapeutic Adherence on the Effectiveness of a Digital Therapeutic Exercise Program: A Propensity Score Matching Analysis
Source: Healthcare (Basel). 2023 Sep 23;11(19):2614. doi: 10.3390/healthcare11192614 (PMC10572588; doi:10.3390/healthcare11192614)
Supplement: Supplementary file 1 [file healthcare-11-02614-s001.zip › Supplementary_4.pdf]

Supplementary File 4: Achievement of clinically relevant improvement of pain intensity, prior to PS-matching for patients with high use intensity (HI), intermediate use intensity (II), low use intensity (LI) and no relevant use intensity (ie, < 1 routine/week, sub-LI). Sensitivity analysis with a threshold of a 1-point VNRS improvement in accordance with Leiva et al. (Ref. 10 in main article).

**Usage Profile**	**Achievement of a clinically relevant improvement of pain intensity at termination of use (%)**
**HI**	59.2
**II**	53.3
**LI**	46.8
**Sub-LI**	41.1
