# Supplementary material for: The Effect of Therapeutic Adherence on the Effectiveness of a Digital Therapeutic Exercise Program: A Propensity Score Matching Analysis
Source: Healthcare (Basel). 2023 Sep 23;11(19):2614. doi: 10.3390/healthcare11192614 (PMC10572588; doi:10.3390/healthcare11192614)
Supplement: Supplementary file 1 [file healthcare-11-02614-s001.zip › Supplementary_5.pdf]

Supplementary File 5: Achievement of clinically relevant improvement of pain intensity, prior to PS-matching for patients with high use intensity (HI), intermediate use intensity (II), low use intensity (LI) and no relevant use intensity (ie, < 1 routine/week, sub-LI). Sensitivity analysis with a threshold of a 2-point VNRS improvement in accordance with Salaffi et al. (Ref. 11 in main article).

**Usage Profile**	**Achievement of a clinically relevant improvement of pain intensity at termination of use (%)**
**HI**	41.3
**II**	35.8
**LI**	27.7
**Sub-LI**	27.1
