# Supplementary material for: The Effect of Therapeutic Adherence on the Effectiveness of a Digital Therapeutic Exercise Program: A Propensity Score Matching Analysis
Source: Healthcare (Basel). 2023 Sep 23;11(19):2614. doi: 10.3390/healthcare11192614 (PMC10572588; doi:10.3390/healthcare11192614)
Supplement: Supplementary file 1 [file healthcare-11-02614-s001.zip › Supplementary_6.pdf]

Supplementary File 6: Differential probability of achieving a clinically relevant improvement of pain intensity at the termination of use, PS-matched analysis for patients with high use intensity (HI), intermediate use intensity (II), low use intensity (LI) and no relevant use intensity (ie, < 1 routine/week, sub-LI). The Bonferroni method was used to adjust the results for multiple testing; significance can be assumed if $P < 0.0167$. Sensitivity analysis with a threshold of a 1-point VNRS improvement in accordance with Leiva et al. (Ref. 10 in main article).

	n	Coefficient	Robust SE	z	P < |z|	95%-CI
HI vs. II/LI/Sub-LI	7,628	0.1	0.0129	7.71	<0.0001	0.074-0.125
II vs. LI/Sub-LI	2,684	0.07	0.021	3.32	0.001	0.028-0.108
LI vs. Sub-LI	1,326	0.07	0.043	1.63	0.104	-0.014-0.156
