# Supplementary material for: The Effect of Therapeutic Adherence on the Effectiveness of a Digital Therapeutic Exercise Program: A Propensity Score Matching Analysis
Source: Healthcare (Basel). 2023 Sep 23;11(19):2614. doi: 10.3390/healthcare11192614 (PMC10572588; doi:10.3390/healthcare11192614)
Supplement: Supplementary file 1 [file healthcare-11-02614-s001.zip › Supplementary_7.pdf]

Supplementary File 7: Differential probability of achieving a clinically relevant improvement of pain intensity at the termination of use, PS-matched analysis for patients with high use intensity (HI), intermediate use intensity (II), low use intensity (LI) and no relevant use intensity (ie, < 1 routine/week, sub-LI). The Bonferroni method was used to adjust the results for multiple testing; significance can be assumed if $P < 0.0167$. Sensitivity analysis with a threshold of a 2-point VNRS improvement in accordance with Salaffi et al. (Ref. 11 in main article).

	n	Coefficient	Robust SE	z	P < |z|	95%-CI
HI vs. II/LI/Sub-LI	7,628	0.1	0.0124	8.48	<0.0001	0.08-0.129
II vs. LI/Sub-LI	2,684	0.09	0.019	4.58	<0.0001	0.049-0.122
LI vs. Sub-LI	1,326	0.11	0.045	0.24	0.814	-0.077-0.099
